# Evaluation and Treatment of Entrapped Peripheral Nerve Catheters: A Case Report and Review

**DOI:** 10.7759/cureus.59487

**Published:** 2024-05-01

**Authors:** Meera Reghunathan, John J Finneran, Brady Huang, Karen Y Cheng, Regine Goh, Katherine Hinchcliff

**Affiliations:** 1 Plastic and Reconstructive Surgery, University of California San Diego, San Diego, USA; 2 Anesthesiology, University of California San Diego, San Diego, USA; 3 Radiology, University of California San Diego School of Medicine, La Jolla, USA; 4 Radiology, University of California San Diego, San Diego, USA

**Keywords:** ultrasound-guided regional anesthesia, iatrogenic complication, peripheral nerve catheters, brachial plexus injury, nerve catheter

## Abstract

Methods to remove retained peripheral nerve catheters range from non-invasive techniques to open surgical procedures. This study reviews two cases requiring surgical intervention for catheter remnant removal after catheter breakage and presents a systematic review describing the diagnosis and treatment of retained perineural catheters. While still very rare, our case report and systematic review demonstrate that retained nerve catheters can occur as the result of kinking or knotting, but also from catheter breakage. We recommend risk mitigation strategies for providers placing or caring for patients with regional nerve catheters.

## Introduction

Continuous peripheral nerve blocks provide postoperative analgesia following painful orthopedic surgery of the extremities. This modality provides superior postoperative analgesia, reduces opioid consumption, and improves patient satisfaction compared to single-injection peripheral nerve blocks or no regional anesthetic [1,2]. A rare complication of perineural catheter insertion is catheter knotting, which can result in an inability to remove the catheter or breakage during attempted removal [3-15].

Previous reports have described various methods to remove retained peripheral nerve catheters. These range from noninvasive techniques, such as positioning changes and saline boluses, to more invasive techniques using fluoroscopically guided instruments or open surgical procedures [3-15]. In this article, we review two cases requiring surgical intervention for catheter remnant removal after catheter breakage and present a systematic review of the published literature regarding the diagnosis and treatment of retained perineural catheters.

## Case presentation

Two patients were identified with retained perineural catheters following breakage during catheter removal. Retrospective data were collected on these patients, including demographics, comorbidities, anesthetic records, medical imaging, and surgical procedure data from the institution’s electronic medical record (EMR).

The University of California, San Diego Institutional Review Board (San Diego, CA) reviewed this study and granted an exemption (Study # 805005). Written consent documenting Health Insurance Portability and Accountability Act authorization and allowing for the publication of non-identifying medical information, photographs, and imaging in the form of a case series was obtained from both patients. This review was not registered. The review protocol can be accessed by contacting the authors.

Case 1: Retained sciatic nerve catheter

Patient Information and Block Placement

A man in his 40s presented to the emergency room with multiple operative lower extremity fractures after sustaining a fall from a significant height. On hospital day four, the patient had bilateral popliteal sciatic non-stimulating continuous catheters (FlexTip Plus, Teleflex Medical, Wayne, PA) placed in the prone position via ultrasound guidance with an in-plane lateral to medial approach as previously described [16]. Following placement, 0.2% ropivacaine was infused via each of the catheters at a continuous rate of 6 milliliters per hour on each side with a 4-milliliter patient-controlled bolus and a 60-minute lockout. The patient had excellent analgesia for the duration of the ropivacaine infusions. Ten days after catheter placement, the anesthesia provider successfully removed the right sciatic catheter. However, when traction was placed on the left sciatic catheter to remove it, the catheter did not exit the patient easily. The provider tried multiple options to facilitate removal, including administering a bolus of saline via the catheter and placing a dilator over the catheter. Despite these maneuvers, the catheter remained stuck in the patient’s leg. A further attempt to place more traction on the catheter ultimately resulted in the catheter breaking inside the patient’s leg with approximately 3 cm of catheter retained in the patient.

Workup and Management

**Figure 1:**
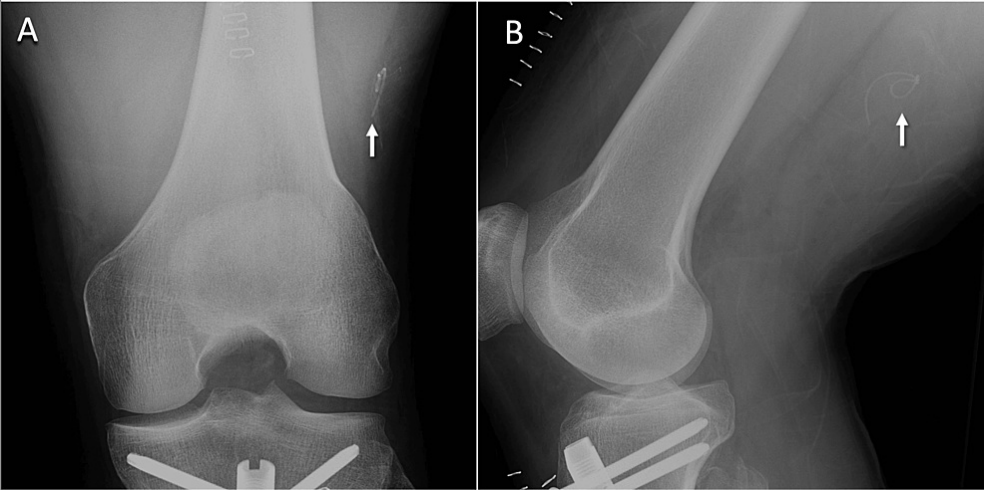
Radiograph of the left thigh showing looped catheter. X-ray imaging of the left thigh is shown in the (A) anteroposterior and (B) lateral view with a white arrow pointing to the retained catheter.

**Figure 2:**
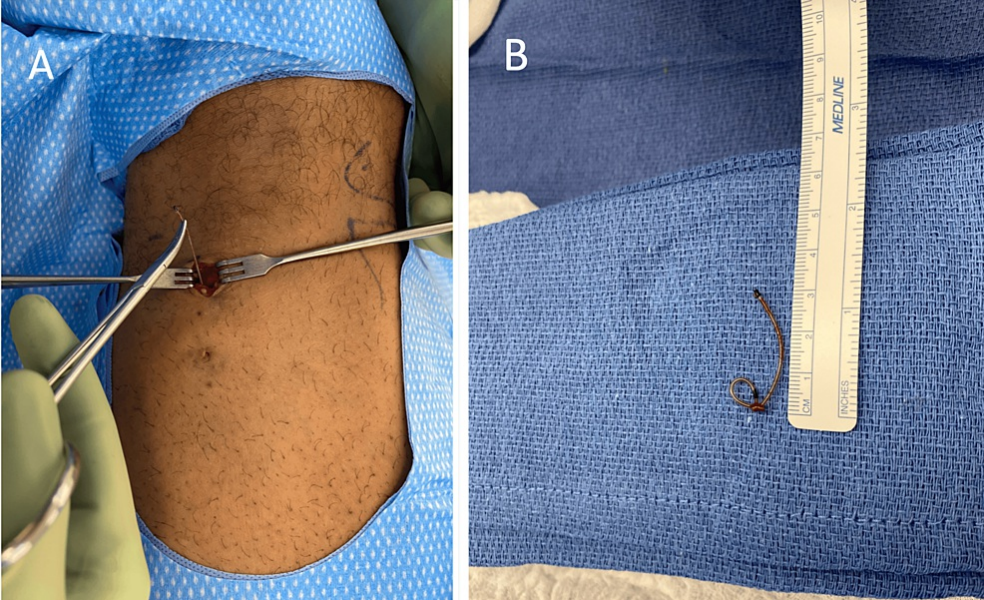
Intraoperative photos of the retained left thigh catheter. The photos show the retained catheter (A) in vivo and (B) knotted as seen ex vivo.

A radiograph of the left knee was obtained, which showed a looped fragment of the broken catheter located in the lateral thigh (Figure 1). The day after the attempted catheter removal, the patient was taken to the operating room by the general surgery team for retrieval of the retained nerve catheter. The anesthesia team used an ultrasound to mark the position of the catheter remnant in concordance with the radiograph. A 1 cm incision was made over the marked area and the catheter was encountered with surgical exploration in the subcutaneous tissue near the fascia of the tensor fasciae latae muscle (Figure 2A). The catheter was easily removed by the surgeon and found to be knotted (Figure 2B). The patient did not complain of any symptoms related to the retained catheter at subsequent follow-up visits.

Case 2: Retained catheter in the infraclavicular brachial plexus

Patient Information and Block Placement

A woman in her late 70s presented with a left olecranon fracture after a ground-level fall and underwent open reduction and internal fixation. Preoperatively, a left infraclavicular non-stimulating continuous nerve catheter (FlexTip Plus, Teleflex Medical, Wayne, PA) was placed using ultrasound guidance via an in-plane parasagittal approach as previously described [17]. Following operative fixation of the fracture, the patient was discharged to her home on the first postoperative day. Following placement, 0.2% ropivacaine was infused via each of the catheters at a continuous rate of 8 milliliters per hour with a 4-milliliter patient-controlled bolus and a 30-minute lockout. She received daily telephone follow-ups to assess the status of her block. The infusion was exhausted at approximately 5:00 AM on the third postoperative day and the catheter was removed by the patient without noted complication. The patient was given the standard postoperative instructions for removal of the catheter on her own, which included diagram depictions and telephone follow-up to ensure the catheter had been removed without issue.

Workup and Management

**Figure 3:**
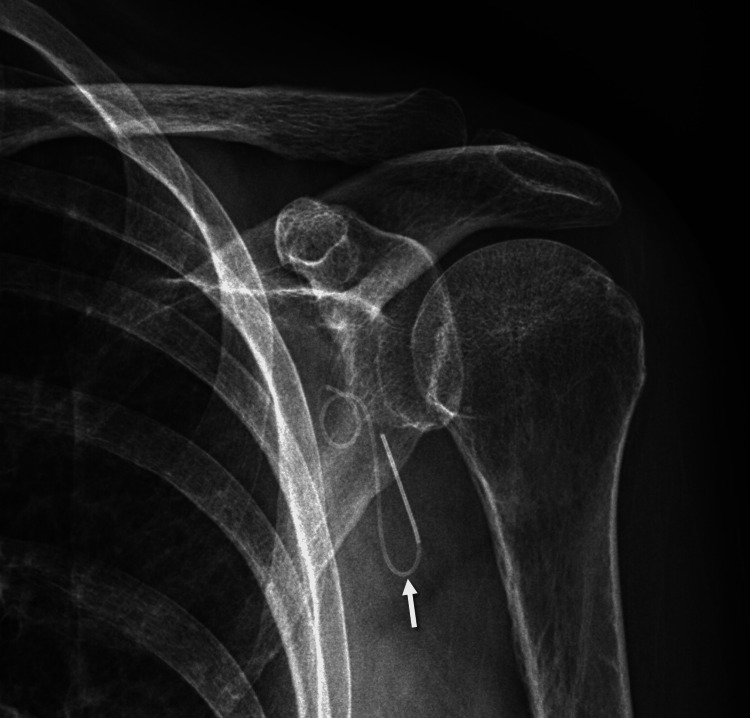
Radiograph of the left shoulder showing the retained catheter. X-ray imaging of the left shoulder in the anteroposterior view is shown with a white arrow pointing to the retained catheter.

**Figure 4:**
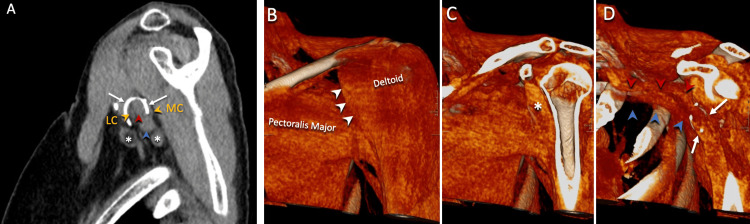
CT scan of the left shoulder. The 3D CT images demonstrated that the denser portion of the catheter, which measures approximately 22 mm in length, resided within the coracobrachialis/short head of the biceps muscle/tendon, the midportion of the catheter looped in between these muscles and the anterior aspect of the axillary neurovascular bundle, and the less radio-dense loop of the catheter remained interposed between the distal brachial plexus/cords and posterior wall of the axillary artery. An axial view (A) is shown demonstrating the lateral cord (LC), medial cord (MC), axillary artery (red carrot), axillary vein (blue carrot), and lymph nodes (white asterisk). The three-dimensional reformatted images (B-D) were particularly helpful in orienting the surgical team to the location of the catheter within the infraclavicular exposure to the brachial plexus. These images demonstrate the axillary artery (red carrot), axillary vein (blue carrot), and short-head biceps/coracobrachialis complex (white asterisk).

Four months after surgery, the patient complained to her primary care provider of dull constant pain over her deltoid and mild shoulder weakness, including trouble opening doors. Deltoid strength was 4+/5 on the modified British Medical Research Council (BMRC) scale, without deltoid atrophy noted on the exam. Her upper extremity motor and sensation exam were otherwise normal. Physical therapy was prescribed for the patient’s rotator cuff symptoms. When three months of therapy failed to improve her symptoms, a shoulder radiograph was obtained, demonstrating a retained catheter tip around the level of her infraclavicular plexus (Figure 3). A subsequent computed tomography (CT) scan confirmed these findings. 3D volume rendered post-processed images from the CT (Impax Volume Viewing 3D software, Agfa HealthCare, Mortsel, Belgium) allowed visualization of the catheter relative to the neurovascular structures of the infraclavicular plexus (Figure 4 and Video 1).

**Video 1:** Three-dimensional CT images in coronal section. This video shows coronal views of the 3D CT images, with the catheter visible between muscle landmarks.

**Figure 5:**
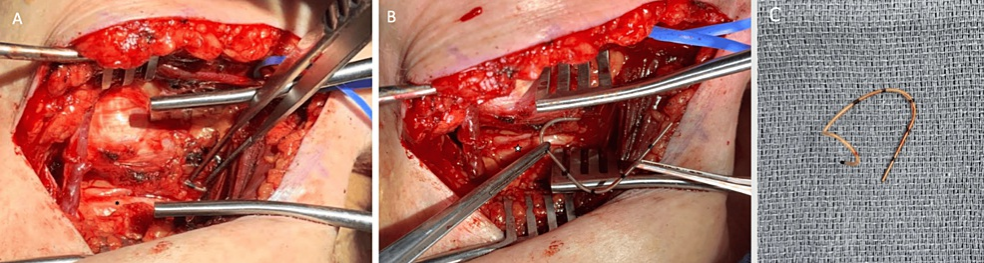
Intraoperative photos of the nerve catheter adjacent to the brachial plexus. Retained catheter being (A) identified and (B) removed from adjacent to the infraclavicular brachial plexus. The vessel loop marks the cephalic vein and the white star designates the lateral cord. In image (C), the retained catheter segment is shown upon explanation.

Using the 3D renderings to guide surgical planning, catheter removal was surgically removed via a standard infraclavicular approach to the brachial plexus. The catheter was encountered as anticipated, posterior to the axillary artery, interposed between the cords of the brachial plexus (Figure 5). The catheter was then removed without difficulty. Minimal scarring and no foreign body reaction were seen around the retained catheter. Postoperatively, the patient wore a shoulder sling for three days and then proceeded with activity as tolerated. At clinic follow-up two months after surgery, the patient reported near resolution of her shoulder pain and on exam had 5/5 strength in her deltoid muscle.

## Discussion

Review of the literature

**Figure 6:**
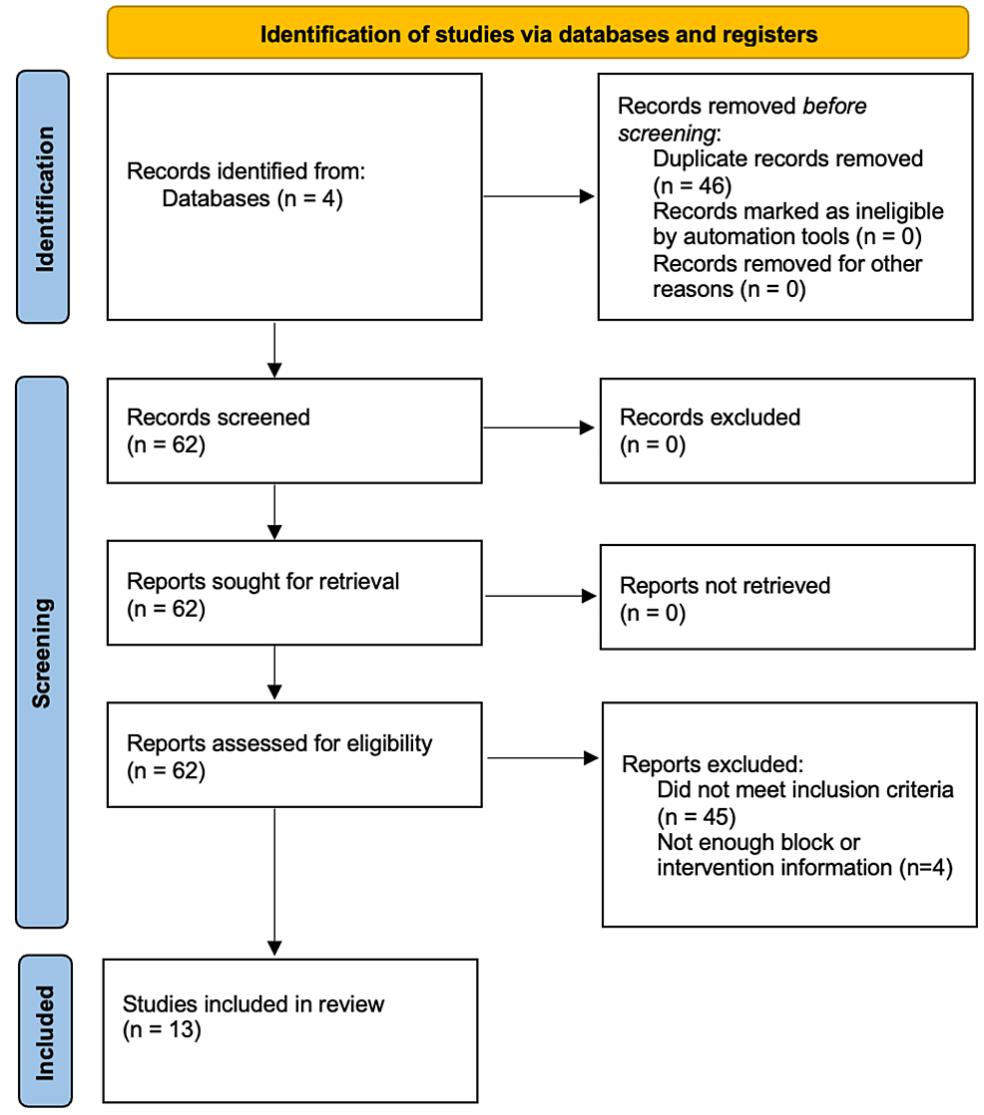
Systematic review search strategy This table summarizes the systematic review search strategy, including which databases were queried and how many articles were excluded.

A systematic review was conducted investigating published workup and treatment strategies for this complication. PubMed, Embase, Cochrane Review, and the National Grey Literature Collection were queried searching for titles or abstracts with the following keywords: (“retained” or “entrapped” or “knotted” or “knotting”) and (“nerve catheter: or “perineural catheter: or “regional anesthesia”). This search initially identified 108 total results (PubMed: 32, Embase: 52, Cochrane Review: 22, and National Grey Literature Collection: 2). After removing duplicates and using the inclusion criteria, 13 individual articles were included in the review (Figure 6). Inclusion criteria were that the article was published between 1990 and 2022, contained at minimum one report of a patient case related to retained perineural catheter with details regarding the technique for catheter retrieval, and was published in English or with an acceptable English translation. Many articles were excluded that mentioned knotting as a potential complication of nerve catheters but did not include any specific case examples. Data were systematically extracted by a single reviewer from these articles to collect details regarding patient demographics, type of block, technique of block insertion, location of entrapment, and technique of retrieval.

Thirteen articles were identified in the systematic review. All were singular case reports, except for two articles, which were case series, with four and eight patients, respectively. Of 23 patients reviewed, nine were femoral, seven were interscalene, one was supraclavicular, one was infraclavicular, one was axillary, two were fascia iliaca, and two were sciatic. Included articles as well as block details are outlined in Table 1. Out of 20 patients for whom these data were specified, 11 patients had the catheter advanced more than 8 centimeters beyond the tip of the needle. In one case, the catheter was threaded as far as 25 centimeters beyond the tip of the needle [5]. The retained catheter was diagnosed due to malfunctioning upon insertion (three patients), upon removal between postoperative days two and 10 (19 patients), or several months following removal due to persistent symptoms (one patient). Five patients in this review attempted the removal of the catheter at home but ultimately presented to the emergency department for further help from a medical provider when they encountered difficulty. Many of the providers in the review attempted positioning changes, saline boluses, and traction. However, these maneuvers were successful for only three patients. Of the remaining 20 patients, 11 patients underwent open surgery for removal, one underwent ultrasound-guided removal, and eight underwent fluoroscopy-guided removal. Table 2 describes further procedural and catheter details. Prior to the procedure, most patients received a plain radiograph, diagnostic ultrasound examination, or both as part of their imaging workup. One patient underwent a CT scan, and one patient underwent a magnetic resonance imaging (MRI) scan. No patients had long-term sequelae or symptoms from their retained nerve catheters or the procedures to retrieve the catheter remnants.

**Table 1:** Catheter and block details for retained nerve catheters in the literature. This table summarizes all articles included in this systematic review. All included articles were either case reports or case series. N/A: information not available; ORIF: open reduction internal fixation; TKA: total knee arthroplasty; I&D: incision and drainage; POD: postoperative day; NS: normal saline.

	First author, year	No. of patients	Indication	Location	Manufacturer	Cm past tip of the needle	How was this detected?	When was this detected?	Anything else attempted to help removal?
1	This study, 2022	2	ORIF olecranon fracture	Infraclavicular	Arrow FlexTip Plus, Teleflex	N/A	Chronic shoulder pain	7 months post-block	N/A
			ORIF multiple lower extremity fractures	Popliteal sciatic	Arrow FlexTip Plus, Teleflex	N/A	Resistance with withdrawal	POD10	Ultrasound, saline bolus, rigid dilator
2	Bowens, 2011 [3]	1	Shoulder arthroscopic surgery	Interscalene	B. Braun	5	Withdrawal of the catheter caused painful paresthesia of ipsilateral deltoid	POD4	An attempt by the anesthesia provider
3	Brenier, 2010 [4]	1	Shoulder arthroscopic surgery	Interscalene	Arrow Stimucath, Teleflex	2	Withdrawal of the catheter caused painful paresthesia in the first and fifth fingers	POD3	An attempt by the anesthesia provider with catheter rotation and sterile NS injection through a catheter
4	Ghanem, 2015 [5]	1	TKA	Femoral	B. Braun	25	Resistance with withdrawal	POD0	N/A
5	Presta, 2012 [6]	1	ORIF olecranon fracture	Supraclavicular	Arrow Stimucath, Teleflex	2-3	Withdrawal of the catheter caused painful paresthesia in the ulnar distribution	POD2	An attempt by the anesthesia provider with positioning, rotation of catheter, and sterile NS injection; interventional radiology attempted to thread the catheter through the Tuohy needle
6	Kendall, 2010 [7]	1	TKA	Femoral	Stimuplex, B. Braun	12	Resistance with withdrawal	POD2	Thigh flexion/ rotation, supine positioning, kept on traction for 6 hours
7	Abrahams, 2011 [8]	4	Shoulder arthroscopic surgery	Interscalene	Stimuplex, B. Braun	4-5	Withdrawal of the catheter caused pain radiating to the thumb and index finger	POD2	An attempt by the anesthesia provider
			ORIF scaphoid	Interscalene	Stimuplex, B. Braun	4-5	Resistance with withdrawal	POD3	Outside emergency department visit
			Shoulder arthroscopic surgery	Interscalene	Stimuplex, B. Braun	4-5	Withdrawal caused numbness in the distal upper extremity and pain radiating to the thumb/index finger	POD0	An attempt by the anesthesia provider with a sterile NS injection
			Shoulder arthroscopic surgery	Interscalene	Stimuplex, B. Braun	4-5	Withdrawal of the catheter with resistance and painful paresthesias radiating to thumb upon attempted removal	POD3	An attempt by the anesthesia provider
8	Khan, 2019 [9]	1	Post-amputation phantom pain	Sciatic	Echogenic Arrow FlexBlock, Teleflex	N/A	Non-healing ulcer at the prior sciatic catheter site, no improvement with antibiotics	5 months post-block	N/A
9	Rogers, 2012 [10]	1	Shoulder arthroscopic surgery	Interscalene	Arrow Stimucath, Teleflex	1	Withdrawal of the catheter caused paresthesias to the thumb	POD3	An attempt by the anesthesia provider with traction
10	Offerdahl, 2004 [11]	1	Total hip arthroplasty	Fascia Iliaca	Portex, Smiths Medical	10	Resistance with withdrawal	POD2	An attempt by the anesthesia provider with positioning
11	Tran, 2005 [12]	1	ORIF radius	Infraclavicular	Stimucath, Arrow Intl	4	Resistance with withdrawal	POD1	N/A
12	Macleod, 2003 [13]	1	I&D knee	Sciatic	B. Braun	N/A	Resistance with withdrawal	POD0	An attempt by the anesthesia provider with positioning, Seldinger technique
13	Rudd, 2004 [14]	1	TKA	Femoral	B. Braun	N/A	Resistance with withdrawal	POD2	An attempt by the anesthesia provider with traction
14	Burgher, 2007 [15]	8	Total hip arthroplasty	Fascia Iliaca	B. Braun	10	Resistance with withdrawal	POD2	An attempt by the anesthesia provider with positioning
			Elbow capsulectomy	Axillary	B. Braun	>8	Resistance with withdrawal	POD5	An attempt by the anesthesia provider with positioning, traction
			TKA	Femoral	B. Braun	10	Resistance with withdrawal	POD2	An attempt by the anesthesia provider with positioning, traction
			TKA	Femoral	B. Braun	10	Resistance with withdrawal	POD4	An attempt by the anesthesia provider with positioning, traction
			TKA	Femoral	B. Braun	10	Resistance with withdrawal	POD3	An attempt by the anesthesia provider with positioning, traction
			TKA	Femoral	B. Braun	10	Resistance with withdrawal	POD3	An attempt by the anesthesia provider with positioning, traction
			TKA	Femoral	B. Braun	10	Resistance with withdrawal	POD3	An attempt by the anesthesia provider with positioning, traction
			TKA	Femoral	B. Braun	8	Resistance with withdrawal	POD3	An attempt by the anesthesia provider with positioning, traction

**Table 2:** Interventions performed for retained nerve catheters in the literature. N/A: information not available; US: ultrasound/ CT: computed tomography scan.

	First author, year	No. of patients	Imaging workup (X-ray, US, CT, MRI)	Intervention	Where was it found?	What was wrong with the catheter?
1	This study, 2023	1	X-ray, CT scan	Surgery	Near axillary artery, between coracobrachialis and biceps as described in CT	N/A
1	Bowens, 2011 [3]	1	N/A	Surgery	Hooked around the C5 nerve root/sheath	The area of focal buckling/kinking caused it to behave like a hook
2	Brenier, 2010 [4]	1	X-ray and US	Surgery	Subcutaneous tissue	A microscopic exam showed major disruption of the flexo-metallic ring
3	Ghanem, 2015 [5]	1	None	Surgery	The catheter passed through the iliopectineal arch and formed a knot dorsal to it (true knot 6 cm away from the catheter tip)	Inserted too far
4	Presta, 2012 [6]	1	X-ray and US	Fluoroscopy-guided removal		The metallic tip extended more than 30 cm beyond the tip of a normal catheter, kinking of the tip
5	Kendall, 2010 [7]	1	US	US-guided removal	Beneath fascia iliaca	Knot at the end of the catheter
6	Abrahams, 2011 [8]	4	X-ray and US	Surgery	Between C5 and C6 nerve roots	N/A
			X-ray	Surgery	N/A	N/A
			None	Non-invasive (saline injection)	N/A	N/A
			X-ray	Surgery	Superior trunk, brachial plexus	Tip deformed but intact
7	Khan, 2019 [9]	1	MRI	Surgery	Between the biceps femoris and vastus muscles	The retained catheter is 15 cm long, the tip was probably not inspected by the ED physician
8	Rogers, 2012 [10]	1	US, CT	Surgery	Adjacent to plexus	Unknown - the company could not determine a design-related cause
9	Offerdahl, 2004 [11]	1	N/A	Non-invasive	N/A	Knot 2 cm from the tip
10	Tran, 2005 [12]	1	N/A	Procedure with a local anesthetic	N/A	The catheter was cut by a nurse and became separated from the wire
11	Macleod, 2003 [13]	1	None	Surgery	Knotted just below the surface of the skin	Excessive feeding of the catheter during needle removal
12	Rudd, 2004 [14]	1	None	Surgery	Knot 1 cm below the skin within the subcutaneous tissue	N/A
13	Burgher, 2007 [15]	8	N/A	Non-invasive (positioning and traction)	N/A	Knot
			N/A	Fluoroscopy-guided removal	N/A	Knot
			N/A	Fluoroscopy-guided removal	N/A	Knot
			N/A	Fluoroscopy-guided removal	N/A	Knot
			N/A	Fluoroscopy-guided removal	N/A	Knot
			N/A	Fluoroscopy-guided removal	N/A	Knot
			N/A	Fluoroscopy-guided removal	N/A	Knot
			N/A	Fluoroscopy-guided removal	N/A	Knot

The reasons for retained catheters

Breakage of perineural catheters during removal most frequently occurs due to a knot in the catheter, which likely occurs as a result of overthreading a catheter via the needle. The coiling of a catheter, which allows for spontaneous knot formation, begins when the catheter has been advanced 3 cm beyond the needle tip [18]. Less commonly, manufacturing defects may result in a weak point in the catheter leading to breakage during removal. Providers (or patients at home) should be instructed on catheter removal and the expected amount of force required. As most catheters have a black or blue tip, written instructions for patients removing catheters at home should specify to look for this tip.

Non-invasive versus surgical management of retained catheters

Identification and removal of retained catheters varies in complexity based on the cause of retention and the anatomic location of the catheter. When the catheter remains transcutaneous but cannot be removed completely, ultrasound imaging can identify the area of snag. Based on our case series and systematic review, this situation is most often related to a looped or knotted catheter that is trapped beneath superficial fascia [4,5]. In these cases, minimally invasive surgical approaches are appropriate. When the retained catheter presents as a deep foreign body, such as the second case in the present series or in the case of Bowens et al. [3], pre-surgical imaging and catheter removal are less straightforward. Most perineural and epidural catheters are compatible with 1.5 and 3.0 Tesla MRI machines; however, the literature is sparse on clear guidance regarding MRI safety [19]. Major concerns related to perineural and epidural catheter exposure to MRI are migration and heating. With countless unique catheters produced by various manufacturers, the limited data available cannot be broadly applied to form a general consensus on perineural catheters' MRI safety. Thus, the risk of catheter migration near critical neurovascular structures may outweigh the benefits of this imaging modality. Three-dimensional rendering of CT images can provide excellent spatial information about the catheter location in relation to surrounding anatomic structures and is our imaging study of choice for pre-surgical planning. Surgical approaches will vary based on the location of the retained catheter, with larger extensile approaches being reserved for cases where dissection around critical neurovascular structures is anticipated.

Limitations

Given the rarity of this nerve catheter complication as well as the sparsity in the literature, all articles included in the systematic review are case reports or case series, representing level IV and V evidence. This limits the ability to draw broad conclusions from the data or perform any meta-analysis to further statistical analysis. All conclusions drawn from the data are based on a small sample size and individual case reports, which cannot account for bias or confounding factors.

## Conclusions

While still very rare, our case report and review of the literature demonstrate that retained nerve catheters can occur as the result of kinking or knotting, but also from catheter breakage. Importantly, the incidence of catheter breakage may be underreported due to delays in recognition of the problem and therefore in presentation and diagnosis.

We recommend multiple risk mitigation strategies for providers placing or caring for patients with regional nerve catheters. If not otherwise limited by the patient's body habitus, never allow the catheter to extend more than 3 cm beyond the needle tip. Provide written or electronic instructions regarding catheter removal that contain pictures of what the catheter tip should look like when removed, or have a mechanism for observed removal by a healthcare provider. If a deep retained catheter is identified and advanced imaging is desired, consider obtaining a CT scan with 3D rendering.
